# Role of MicroRNAs in TGF-β Signaling Pathway-Mediated Pulmonary Fibrosis

**DOI:** 10.3390/ijms18122527

**Published:** 2017-11-25

**Authors:** Hara Kang

**Affiliations:** Division of Life Sciences, College of Life Sciences and Bioengineering, Incheon National University, Incheon 406-772, Korea; harakang@inu.ac.kr; Tel.: +82-32-835-8238; Fax: +82-32-835-0763

**Keywords:** pulmonary fibrosis, TGF-β signaling pathway, microRNAs

## Abstract

Pulmonary fibrosis is the most common form of interstitial lung disease. The transforming growth factor-β (TGF-β) signaling pathway is extensively involved in the development of pulmonary fibrosis by inducing cell differentiation, migration, invasion, or hyperplastic changes. Accumulating evidence indicates that microRNAs (miRNAs) are dysregulated during the initiation of pulmonary fibrosis. miRNAs are small noncoding RNAs functioning as negative regulators of gene expression at the post-transcriptional level. A number of miRNAs have been reported to regulate the TGF-β signaling pathway and consequently affect the process of pulmonary fibrosis. A better understanding of the pro-fibrotic role of the TGF-β signaling pathway and relevant miRNA regulation will shed light on biomedical research of pulmonary fibrosis. This review summarizes the current knowledge of miRNAs regulating the TGF-β signaling pathway with relevance to pulmonary fibrosis.

## 1. Introduction

Pulmonary fibrosis is a chronic, progressive, and fatal lung disease [1]. It is characterized by excessive secretion of pro-fibrotic mediators, such as transforming growth factor-β1 (TGF-β1); aberrant activation of epithelial mesenchymal transition (EMT); activation and proliferation of fibroblasts; persistence of apoptotic resistant myofibroblasts; and recruitment of inflammatory cells in the lesions. TGF-β is the main cytokine implicated in the pathogenesis of pulmonary fibrosis [2]. Due to the absence of effective treatments for reversing the disease or for consistently attenuating fibrotic progression, understanding the mechanisms for fine-tuning of TGF-β signaling pathway and developing strategies to inhibit TGF-β signaling pathway are essential. Indeed, attempts to ubiquitously block TGF-β have been evaluated in mice and humans (available online: http://www.clinicaltrials.gov) [3].

Intensive studies during the past few years have highlighted the role of microRNAs (miRNAs) in lung fibrosis pathophysiology [4,5]. In vivo studies demonstrate that supplementing decreased miRNAs or inhibiting increased miRNAs in bleomycin-induced lung fibrosis is sufficient to either prevent or reverse lung fibrosis [6,7,8,9]. Bleomycin-induced pulmonary fibrosis has been widely used and is a well-characterized model in rodents to elucidate the molecular mechanisms involved in fibrogenesis and for the evaluation of potential therapies [10].

In this review, we summarize the roles of miRNAs that contribute to the pathology observed in pulmonary fibrosis by regulating the TGF-β signaling pathway.

## 2. Transforming Growth Factor-β (TGF-β) Signaling Pathway

TGF-β is one of the most studied pro-fibrotic cytokines. In lungs, TGF-β is produced by a wide variety of cell types, including alveolar macrophages, neutrophils, activated alveolar epithelial cells, endothelial cells, fibroblasts, and myofibroblasts. Upon binding with TGF-β, type II TGF-β receptor (TGF-βRII) recruits type I TGF-β receptor (TGF-βRI) and phosphorylates TGF-βRI, which in turn initiates signal transduction mediated by the downstream Smad proteins [11]. Activated TGF-βRI phosphorylates cytoplasmic Smad2 and Smad3 (Smad2/3) transcription factors, allowing them to translocate into the nucleus. Smad4 acts as a partner with Smad2/3 to facilitate this process. This translocated heteromeric complex modulates pro-fibrotic genes, promoting the differentiation of fibroblasts to myofibroblasts such as α-smooth muscle actin (α-SMA), inducing EMT, and enhancing the deposition of collagen [12]. The activated TGF-β signaling pathway also stimulates the expression of a number of proinflammatory and fibrogenic cytokines, such as tumor necrosis factor-α (TNF-α), interleukin-1 β (IL-1b), IL-13, or platelet-derived growth factor (PDGF), thereby further enhancing and perpetuating the fibrotic response. On the other hand, the TGF-β signaling pathway induces the expression of inhibitory Smads, Smad6 and Smad7, as a part of a negative feedback loop. Smad6 and Smad7 antagonize activation of receptor-regulated Smad 2/3 by competing with Smad4 or TGF-βRI. The stability of Smad2/3 is regulated by Smurf2 E3 ubiquitin ligase. Therefore, the TGF-β signaling pathway is downregulated by Smad6, Smad7, and Smurf2 [11].

## 3. MicroRNAs

miRNAs are evolutionarily conserved short noncoding RNAs that regulate post-transcriptional mRNA expression by binding to target mRNAs, resulting in gene silencing via translational repression and/or destabilization of mRNA [13]. miRNAs function by partially complementary base pairing with the 3′-untranslated regions of target mRNAs.

miRNAs have been shown to control various cellular processes such as differentiation, proliferation, and cell-cell interaction [14]. In addition, the dysregulation of miRNAs is linked to a variety of diseases, including proliferative vascular disease, cardiac disorders, fibrosis, and cancer [15,16,17,18]. Recent studies of miRNA expression identified a significant change in 10% of miRNAs between control and pulmonary fibrosis lungs [19]. Several dysregulated miRNAs have been studied in human lungs with pulmonary fibrosis as well as in animal models of pulmonary fibrosis. These dysregulated miRNAs result in the activation of resident fibroblasts and differentiation to myofibroblasts, proliferation and migration of fibroblasts, aberrant wound healing, and excessive accumulation of extracellular matrix (ECM) [18,20].

**Figure 1:**
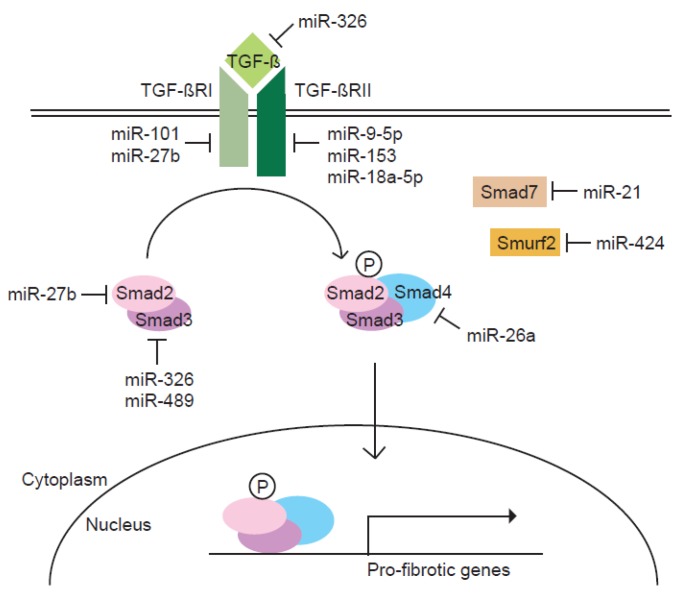
Regulation of the TGF-β signaling pathway by miRNAs. In response to TGF-β signal, the expression of a cohort of miRNAs is modulated. Each miRNA is implicated in the process of pulmonary fibrosis by targeting components of the TGF-β signaling pathway. Normal arrows represent activation and nuclear translocation of Smads. T-bar arrows represent inhibition of indicated proteins.

Most components of the TGF-β signaling pathway are known to be targeted by one or more miRNAs (Figure 1). Since the TGF-β signaling pathway is the primary fibrogenic mediator, the regulation of TGF-β signaling molecules by miRNAs appear to influence the pathogenesis of pulmonary fibrosis. Therefore, this review focuses on roles of miRNAs in regulating the TGF-β signaling pathway in the process of pulmonary fibrosis.

## 4. MicroRNA-Mediated Regulation of the TGF-β Signaling Pathway in Pulmonary Fibrosis

A number of studied microRNAs are linked to the TGF-β signaling pathway by targeting components of TGF-β signaling (Table 1). Emerging studies about their regulation of the TGF-β signaling pathway have shown the role of miRNAs in pulmonary fibrosis. Altering the expression of pro-fibrotic miRNAs or introducing anti-fibrotic miRNAs may result in reversal of the pro-fibrotic milieu in the pulmonary fibrosis.

### 4.1. Pro-Fibrotic MicroRNAs

TGF-β induces miRNAs such as miR-21 and miR-424 that target negative regulators of the TGF-β signaling pathway to amplify the pathway, leading to pulmonary fibrosis.

#### 4.1.1. miR-21

miR-21 appears to be a pro-fibrotic miRNA that amplifies the TGF-β signaling pathway and promotes fibrotic lung diseases including idiopathic pulmonary fibrosis (IPF) [8]. A recent study reported that miR-21 was increased in the lungs of mice with bleomycin-induced lung fibrosis and in the lungs of patients with IPF. The enhanced expression of miR-21 is primarily located in the myofibroblasts of fibrotic lungs. The overexpression of miR-21 promoted the development of bleomycin-induced pulmonary fibrosis, whereas the inhibition of miR-21 in bleomycin-treated lungs reduced the severity of fibrosis and myofibroblast differentiation. Treatment with miR-21 antisense prevented the enhanced collagen deposition and elevated expression of ECM proteins, such as Fn and collagen, in the lungs after bleomycin administration [8].

We have previously shown that miR-21 is induced in TGF-β–treated human vascular smooth muscle cells, which regulate the expression of genes involved in the contraction of smooth muscle cells [28,29]. Similar to the previous study, miR-21 was upregulated by TGF-β and enhanced the fibrogenic activity of the TGF-β signaling pathway in human primary fibroblasts.

Liu et al. identified Smad7, an inhibitory Smad, as an miR-21 target, which was validated by luciferase assay [8]. Smad7 expression was decreased in bleomycin-treated lungs, concurrently with enhanced miR-21 expression under these conditions. Therefore, miR-21, as a pro-fibrotic miRNA, targets Smad7 to enhance the TGF-β signaling pathway and in turn promote fibrotic lung diseases [8].

An miRNA microarray of radiation-induced lung fibrosis demonstrated that miR-21 expression is induced in injured lung tissue [30]. The increase of miR-21 level was correlated with the level of collagen expression, which is a typical phenotype of fibrosis. This suggests the pro-fibrotic function of miR-21 in radiation-induced lung fibrosis as well as bleomycin-induced lung fibrosis.

Recently, it has been shown that baicalein (5,6,7-trihydroxyflavone), a natural plant flavone isolated from the roots of *Scutellaria baicalensis*, can attenuate bleomycin-induced pulmonary fibrosis by regulating miR-21 expression [31]. Baicalein decreased miR-21 levels and inhibited the increased expression of TGF-β and phosphorylated Smad2/3 in bleomycin-treated rats. In addition, radix puerariae extracts were shown to attenuate paraquat-induced lung fibrosis by inhibiting miR-21 and phosphorylated Smad2/3 expression in lungs from paraquat-treated mice [32]. These results demonstrate that miR-21 is a critical pro-fibrotic miRNA and its modulation may have effects on the pathogenesis of pulmonary fibrosis.

#### 4.1.2. miR-424

An miRNA microarray analysis on a human lung epithelial cell model of EMT was performed [21]. During EMT, miR-424 was upregulated and increased miR-424 induced the expression of α-SMA, a marker for myofibroblast differentiation [21]. In lung fibrotic diseases, the persistence of myofibroblasts is an important indicator of the end stage of the disease [33].

miR-424 expression was increased by TGF-β, and miR-424 enhanced the activity of the Smad-dependent TGF-β signaling pathway, as revealed by a TGF-β signaling reporter assay. Smurf2, a negative regulator of the TGF-β signaling pathway, was identified as a target of miR-424. Therefore, miR-424 is a pro-fibrotic miRNA that regulates the myofibroblast differentiation during EMT by potentiating the TGF-β signaling pathway.

### 4.2. Anti-Fibrotic MicroRNAs

Anti-fibrotic miRNAs attenuate the pro-fibrotic function of the TGF-β signaling pathway by suppressing several essential components of the TGF-β signaling pathway [6,9,24,26].

#### 4.2.1. miR-101

Global miRNA expression profiles of the lung tissues from 28 IPF patients obtained from the Lung Tissue Research Consortium (LTRC) were analyzed [7]. As a result, expression levels of miR-101 were significantly reduced. In the lung tissue from an experimental mouse model of bleomycin-induced pulmonary fibrosis, miR-101a expression was also reduced. The overexpression of miR-101 in LL29 IPF fibroblasts inhibited TGF-β-induced protein and mRNA expression of α-SMA and collagens, alpha-1 type I collagen (COL1A1) and alpha-1 type III collagen (COL3A1). Consequently, miR-101 reduced TGF-β1-induced contractile activity as determined by the collagen gel assay and stress fiber formation. In contrast, anti-miR-101 increased the expression of α-SMA and the collagens in CCD-8Lu normal lung fibroblasts. Furthermore, adenovirus-mediated *miR-101* gene transfer in the mouse lung attenuated bleomycin-induced lung fibrosis and improved lung function. Huang et al. identified TGF-βRI as a target of miR-101. All these results demonstrated that miR-101, as an anti-fibrotic miRNA, suppressed the TGF-β-induced activation of lung fibroblasts by the inhibition of Smad2/3 signaling via targeting TGF-βRI [7].

#### 4.2.2. miR-9-5p

TGF-β induced miR-9-5p expression in human fetal lung fibroblasts [6]. Increased levels of miR-9-5p reduced the migration and invasion of ECM in response to TGF-β. Orotracheal lenti-miR-9-5p instillation showed an attenuation of bleomycin-induced lung fibrosis in mice. Similarly, pre-treatment with lenti-miR-9-5p prevented the accumulation of myofibroblasts in bleomycin-treated lungs, as demonstrated by immunohistochemistry staining with α-SMA antibody. In contrast, orotracheal miR-9-5p inhibitor instillation enhanced the accumulation of myofibroblasts in bleomycin-treated lungs. In vitro, the overexpression of miR-9-5p in lung fibroblasts prevented myofibroblast differentiation, activation, migration, and invasion by inhibiting the Smad-dependent TGF-β signaling pathway. Smad2 phosphorylation, Smad3/4 activation, and Smad2/3 nuclear translocation were delayed in lung fibroblasts with increased levels of miR-9-5p. TGF-βRII was identified as a target of miR-9-5p and exogenous expression of TGF-βRII attenuated the inhibitory effect of miR-9-5p on TGF-β-induced Smad2 phosphorylation.

This study suggests an autoregulatory feedback loop between TGF-β and miR-9-5p. TGF-β might trigger both pro- and anti-fibrotic signals. As an anti-fibrotic signal, miR-9-5p is induced by TGF-β and may limit fibrogenesis by counteracting the pro-fibrotic function elicited by the TGF-β signaling pathway in the context of a homeostatic response.

#### 4.2.3. miR-153

Liang et al. showed that miR-153 was dysregulated in the mouse model by intratracheal instillation of bleomycin via miRNA microarrays [23]. When miR-153 was overexpressed in MRC-5 lung fibroblasts, the phosphorylation of Smad2/3 was significantly reduced, and consequently the contractile and migratory activities of lung fibroblasts were attenuated. In contrast, the knock-down of miR-153 promoted the pro-fibrogenic activity of the TGF-β signaling pathway. Moreover, Liang et al. observed that TGF-β1 decreased miR-153 expression and miR-153 targets TGF-βRII in pulmonary fibroblasts [23]. All these data suggest that the autoregulatory loop of TGF-β-miR-153-TGF-βRII is a molecular mechanism for the maintenance of the TGF-β signaling pathway, contributing to the fibrotic response in pulmonary fibrosis [23]. The downregulation of miR-153 by TGF-β signaling might lead to the derepression of TGF-βRII expression, which in turn would augment the pro-fibrotic activity of the TGF-β signaling pathway.

#### 4.2.4. miR-1343

Unlike most previous reports that identified miRNAs involved in pathways of fibrosis through comparisons of normal and fibrotic tissue by differential gene expression, Stolzenburg et al. identified miR-1343 by searching for miRNAs that could directly target TGF-β receptors, both TGF-βRI and TGF-βRII [22]. miR-1343 reduced the phosphorylation of Smad2/3, correlated with a lack of their translocation to the nucleus. In addition, the overexpression of miR-1343 repressed intracellular α-SMA expression. These results suggest that miR-1343, as an anti-fibrotic miRNA, can repress phenotypes associated with the TGF-β-induced transition of resident fibroblasts to myofibroblasts by directly targeting TGF-βRI and TGF-βRII.

#### 4.2.5. miR-18a-5p

Zhang et al. observed that the inhibition of miR-18a-5p results in murine pulmonary fibrosis [9]. Mice treated with intraperitoneal injection of a lentivirus encoding siRNA directed against miR-18a-5p showed induced sub–pleural fibrosis. Levels of phosphorylated Smad2/3 were increased in lung tissues of miR-18a-5p siRNA-treated mice compared with control mice. On the other hand, when lentivirus expressing miR-18a-5p was injected intraperitoneally into mice after bleomycin administration, pulmonary fibrosis and sub-pleural fibrosis were attenuated. In this study, the role of miR-18a-5p was also investigated in EMT of pleural mesothelial cells (PMCs). Inhibition of miR-18a-5p using lentivirus-mediated miR-18a-5p siRNA induced EMT of PMCs and promoted PMC migration. This inhibition of miR-18a-5p was associated with an increase in phosphorylated Smad2/3 levels in PMCs. In contrast, the overexpression of miR-18a-5p prevented bleomycin–induced EMT and the cell migration of PMCs.

Zhang et al. determined that miR-18a-5p targets TGF-βRII, and this is associated with suppression of the TGF-β signaling pathway [9]. Therefore, miR-18a-5p might negatively regulate the TGF-β signaling-mediated EMT pathway in PMCs and a pulmonary fibrosis animal model by targeting *TGF-βRII* gene expression. 

#### 4.2.6. miR-326 

miR-326 has been shown to regulate TGF-β1 expression, and miR-326 levels are inversely correlated to TGF-β1 levels in multiple human cell lines [24]. Das et al. investigated the anti-fibrotic role of miR-326. They observed that miR-326 was downregulated in patients with IPF and during the progression of bleomycin-induced pulmonary fibrosis in mice. Moreover, TGF-β levels and collagen content were found to be significantly higher in patients with IPF as compared to non-fibrotic controls.

Ectopic expression of miR-326 was able to rescue epithelial cells from EMT and attenuated fibrotic pathogenesis. Smad3 was downregulated in miR-326-transfected cells and validated as a target of miR-326. Conversely, treatment with anti–miR-326 promoted EMT and enhanced TGF-β production. Moreover, in vivo inhaled delivery of hairpin nucleotides mimicking miR-326 was sufficient to inhibit TGF-β1 expression and attenuate the fibrotic process. All these data suggest that miR-326 plays an anti-fibrotic role by inhibiting the TGF-β signaling pathway through the direct targeting of TGF-β and Smad3.

#### 4.2.7. miR-27b

miR-27b was identified as a major miRNA in modulating TGF-β-induced collagen I expression using an miRNA inhibitor library [25]. The expression of miR-27b in the lung tissue of bleomycin-treated mice was decreased compared to that of the control mice, and the treatment of bleomycin reduced miR-27b expression in the fibroblasts. This suggests that miR-27b is associated with fibrotic lungs. The overexpression of miR-27b in LL29 human pulmonary fibroblasts using a lentiviral vector inhibited the TGF-β-induced contractility of LL29 fibroblasts. In addition, exogenous miR-27b decreased collagen synthesis and inhibited the expression of α-SMA, suggesting that miR-27b suppresses TGF-β-induced fibroblast activation. TGF-βRI and Smad2 were identified as targets of miR-27b. Therefore, miR-27b is an anti-fibrotic miRNA in pulmonary fibroblasts that inhibits fibroblast activation by the repression of direct targets, such as TGF-βRI and Smad2.

#### 4.2.8. miR-26a

miR-26a was downregulated in the lungs of IPF patients and in mice with experimental pulmonary fibrosis [27]. Interestingly, the downregulation of miR-26a was caused by the TGF-β-mediated phosphorylation of Smad3. Analysis of the putative promoter sequence by the Genomatix algorithm revealed a binding site for Smad3 in the upstream region of the *miR-26a* gene, suggesting a novel autoregulatory loop between miR-26a and phosphorylated Smad3 in the context of pulmonary fibrosis. AntagomiR-26a caused a marked collagen deposition, which was similar to that caused by bleomycin. Similarly, the transfection of the miR-26a inhibitor induced significant upregulation of COL1A1 and COL3A1 in MRC-5 cells. These results suggest that both in vivo and in vitro inhibition of miR-26a in the lung causes pulmonary fibrosis. Smad4, a co-Smad that plays an essential role in nuclear translocation of phosphorylated Smad2/3, was identified as a target of miR-26a. The overexpression of miR-26a inhibited the nuclear translocation of phosphorylated Smads by directly targeting Smad4, which blocked signaling events downstream of TGF-β and finally alleviated collagen deposition and reduced the lung fibrosis. All these data demonstrate that TGF-β modulates the expression of anti-fibrotic miR-26a to maintain the pro-fibrotic functions of the TGF-β signaling pathway.

#### 4.2.9. miR-489

Wu et al. observed that miR-489 levels were decreased in a mouse model of silica-induced pulmonary fibrosis by miRNA microarray analyses [26]. When miR-489 agomir was injected via intratracheal instillation after silica treatment, less severe fibrotic foci and less destruction of alveolar architecture were observed, demonstrating an anti-fibrotic function of miR-489 in silica-induced pulmonary fibrosis.

Interestingly, the expression level of long noncoding RNA CHRF is increased in silica-induced pulmonary fibrosis. CHRF has been reported to be an endogenous “sponge” of miR-489 that inhibits the repression of miR-489 on its target genes in cardiac hypertrophy [34]. Wu et al. demonstrated that Smad3 is a target of miR-489 in fibroblasts. Therefore, the induced CHRF might inhibit miR-489 expression and consequently derepress the target genes, Smad3, to maintain the TGF-β signaling pathway in silica-induced pulmonary fibrosis.

## 5. Discussion and Conclusions

miRNAs have been considered to contribute to default repression. Default repression ensures that a target gene expression is turned on exclusively in the presence of signaling cues. Thus, miRNAs affect the responsiveness of cells to signaling molecules such as TGF-β. Upon signaling cues, miRNAs amplify signals through the coordinated regulation of multiple targets. The multi-gene regulatory capacity of miRNAs facilitates the transmission of information to downstream molecules in an effective and timely manner. The fact that a single miRNA such as miR-23b targets several Smads explains how the simultaneous targeting of miRNAs on a common set of regulatory proteins can amplify their effect, even though they have a weak effect on their own. If an miRNA targets an inhibitor of a signaling pathway, it serves as a positive regulator by either amplifying signal strength or duration. Many signaling pathways are specifically involved in disease processes. Aberrant expression of miRNAs plays a pivotal role in developmental defects and the pathogenesis of diseases.

To date, there have been numerous efforts to identify dysregulated miRNAs and characterize their functions in the process of pulmonary fibrosis. Given the importance of the TGF-β signaling pathway in the development of pulmonary fibrosis, relevant miRNAs that modulate the TGF-β signaling pathway were highlighted as possible new targets to develop efficient therapies for pulmonary fibrosis. The identification of more relevant miRNAs will likely increase the use of miRNAs as therapeutic targets for pulmonary fibrosis. The simple inactivation of an miRNA may not be sufficient, but administering oligonucleotides against multiple miRNAs could be effective in the same way that a cocktail of small-molecule drugs can have more enhanced therapeutic effect than any of the drugs alone. Simultaneous manipulation of more than one miRNA may prove beneficial for additive effects.

**Figure 2:**
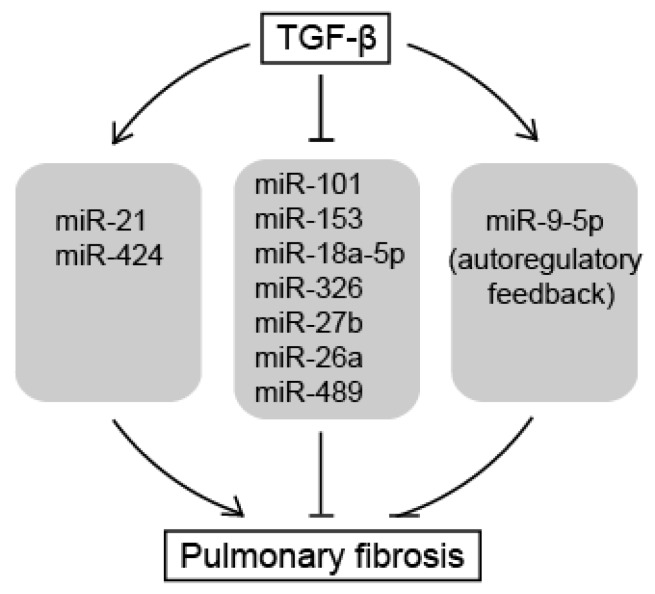
miRNAs as downstream effectors of TGF-β signaling in pulmonary fibrosis. TGF-β amplifies TGF-β signals by repressing anti-fibrotic miRNAs as well as inducing pro-fibrotic miRNAs, resulting in pulmonary fibrosis. Normal arrows represent upregulation of miRNA expression or promotion of pulmonary fibrosis. T-bar arrows represent downregulation of miRNA expression or inhibition of pulmonary fibrosis.

In this review, we summarized the roles of miRNAs as downstream effectors of TGF-β signaling pathways that induce pulmonary fibrosis (Figure 2). Pro-fibrotic miRNAs, such as miR-21 and miR-424, are induced by TGF-β signals and potentiate the TGF-β signaling pathway to promote the phenotype of pulmonary fibrosis by repressing negative regulators of the TGF-β signaling pathway. In contrast, anti-fibrotic miRNAs, such as miR-101, miR-18a-5p, miR-1343, miR-9-5p, miR-153, miR-326, miR-27b, miR-489, and miR-26a, inhibit the TGF-β signaling pathway by targeting positive regulatory molecules of the signaling and attenuate the development of pulmonary fibrosis. They are downregulated in the pathogenic lungs, apart from miR-9-5p. miR-9-5p plays a role as an anti-fibrotic miRNA, but is induced by TGF-β to form an autoregulatory feedback loop. Taken together, this suggests that many anti-fibrotic miRNAs contribute to default repression at the level of downstream target of the TGF-β signaling pathway for the prevention of pulmonary fibrosis. To initiate pulmonary fibrosis, TGF-β amplifies TGF-β signals by repressing the anti-fibrotic miRNAs as well as inducing pro-fibrotic miRNAs. However, the factors that regulate these miRNAs and the mechanisms by which miRNAs are being down- or upregulated during pulmonary fibrosis still needs to be investigated. The identification of the genetic or epigenetic elements responsible for the regulatory mechanisms of miRNA expression is essential to dissect the role of miRNAs in signaling networks.

Using oligonucleotides in the treatment of diseases requires the development of a novel methodology for the more efficient and targeted delivery of oligonucleotides to specific target tissues. Studies on circulating miRNAs and on the mechanism of selective miRNA uptake by target tissues may contribute to the development of a more effective delivery method of miRNAs or antisense RNA oligonucleotides against miRNAs. Recently, Huang et al. reported that miR-125b-5p, miR-128, miR-30e, and miR-20b were significantly altered in the lung tissue and plasma of smoking-induced pulmonary fibrosis [35]. Serum miR-21 was also reported to be highly expressed in IPF patients compared to healthy people, and was associated with the severity of tissue damage [36]. Studies on these circulating miRNAs would provide invaluable information for developing diagnostic biomarkers for pulmonary fibrosis as well as developing more effective delivery methods. Further investigation of their crosstalk with the TGF-β signaling pathway would be also interesting.

## 6. Summary Points

- A number of miRNAs are involved in the TGF-β signaling pathway and consequently affect the process of pulmonary fibrosis.
- TGF-β amplifies TGF-β signals by repressing anti-fibrotic miRNAs as well as inducing pro-fibrotic miRNAs, resulting in pulmonary fibrosis.
- The identification of more relevant miRNAs will increase the use of miRNAs as therapeutic targets or diagnostic tools for pulmonary fibrosis.

## Figures and Tables

**Table 1:** miRNAs regulating the TGF-β signaling pathway.

**Pro-Fibrotic miRNAs**		
**miRNA**	**Validated Targets**	**Reference**
miR-21	Smad7	Liu et al., 2010 [8]
miR-424	Smurf2	Xiao et al., 2015 [21]
**Anti-Fibrotic miRNAs**		
miR-101	TGF-βRI	Huang et al., 2017 [7]
miR-18a-5p	TGF-βRII	Zhang et al., 2017 [9]
miR-1343	TGF-βRI, TGF-βRII	Stolzenburg et al., 2016 [22]
miR-9-5p	TGF-βRII	Fierro-Fernández et al., 2015 [6]
miR-153	TGF-βRII	Liang et al., 2015 [23]
miR-326	TGF-β1, Smad3	Das et al., 2014 [24]
miR-27b	TGF-β1, Smad2	Zeng et al., 2017 [25]
miR-489	Smad3	Wu et al., 2016 [26]
miR-26a	Smad4	Liang et al., 2014 [27]
